# The electronic properties of SrTiO_3-δ_ with oxygen vacancies or substitutions

**DOI:** 10.1038/s41598-021-02751-9

**Published:** 2021-12-02

**Authors:** L. L. Rusevich, M. Tyunina, E. A. Kotomin, N. Nepomniashchaia, A. Dejneka

**Affiliations:** 1 Institute of Solid State Physics, University of Latvia, Kengaraga Str. 8, Riga, 1063 Latvia; 2 Microelectronics Research Unit, Faculty of Information Technology and Electrical Engineering, University of Oulu, P. O. Box 4500, 90014 Oulu, Finland; 3 Institute of Physics of the Czech Academy of Sciences, Na Slovance 2, 18221 Prague, Czech Republic; 4 Max Planck Institute for Solid State Research, Heisenberg Str. 1, 70569 Stuttgart, Germany

**Keywords:** Electronic properties and materials, Ferroelectrics and multiferroics, Surfaces, interfaces and thin films

## Abstract

The electronic properties, including bandgap and conductivity, are critical for nearly all applications of multifunctional perovskite oxide ferroelectrics. Here we analysed possibility to induce semiconductor behaviour in these materials, which are basically insulators, by replacement of several percent of oxygen atoms with nitrogen, hydrogen, or vacancies. We explored this approach for one of the best studied members of the large family of *ABO*_*3*_ perovskite ferroelectrics — strontium titanate (SrTiO_3_). The atomic and electronic structure of defects were theoretically investigated using the large-scale first-principles calculations for both bulk crystal and thin films. The results of calculations were experimentally verified by studies of the optical properties at photon energies from 25 meV to 8.8 eV for in-situ prepared thin films. It was demonstrated that substitutions and vacancies prefer locations at surfaces or phase boundaries over those inside crystallites. At the same time, local states in the bandgap can be produced by vacancies located both inside the crystals and at the surface, but by nitrogen substitution only inside crystals. Wide-bandgap insulator phases were evidenced for all defects. Compared to pure SrTiO_3_ films, bandgap widening due to defects was theoretically predicted and experimentally detected.

## Introduction

Strontium titanate (SrTiO_3_, or STO) is one of the best studied materials belonging to a broad class of perovskite-structure oxide ferroelectrics^1^. Spontaneous polarization, large dielectric permittivity, strong piezoelectric, pyroelectric, electrocaloric, photovoltaic, and electro-optic effects enable numerous applications of ferroelectrics in diverse fields of electronics and photonics^1–8^. Requirements to the electronic properties are application-specific and vary significantly. For instance, perfect insulator properties are demanded for majority of the mainstream ferroelectric applications (e.g., in capacitors, electro-mechanical, and electro-optical devices), whereas bandgap narrowing and increased semiconductor- or metal-like conductivity are desirable for innovative photovoltaic, thermoelectric, and resistive-switching devices. Remarkably, such immense variations of the electronic properties are feasible using appropriate doping within the class of perovskite oxide ferroelectrics. However, although there are successful practical strategies, explicit mechanisms of the properties’ changes are far from understood. In particular, one of the methods relies on the possibility to remove oxygen atoms and/or substitute oxygen with hydrogen or nitrogen^9–16^. Compared to a wide-bandgap insulator state in pure crystalline STO, semiconductor-type behaviour was reported for SrTiO_3-x_H_x_ (x = 0.05–0.45) and SrTiO_3-x_N_x_ (x = 0.20)^10,15,16^. Substitution-induced shallow donor states were hypothesized to be responsible for the bandgap narrowing and large band conductivity therein. Yet, this hypothesis has not been proven.

It is worth mentioning that the removal and/or replacement of oxygen is often implemented by high-temperature processing that can last for several hours up to several days^10,11^. Under such conditions, one cannot exclude the formation of local compositional and/or structural inhomogeneities and, correspondingly, different types of phase boundaries, or interfaces. Importantly, the presence of interfaces is known to alter the properties of regular perovskite oxide ferroelectrics^17,18^. This presence additionally suggests the coexistence of two distinct types of substitutional locations: inside single crystal and at interfaces. The location may have an influence on the electronic structure and properties of STO. Furthermore, high-temperature processing may generate structural defects, such as dislocations^19^, whose role may be essential.

In this paper, we combined theoretical and experimental methods to elucidate the electronic properties, that originate from substitutions for oxygen either inside crystal or at interfaces in STO. Substitution of several percent of oxygen atoms with nitrogen, hydrogen, or vacancies was investigated.

We theoretically analysed crystalline STO with substitutions/vacancies inside the crystal. Because of uncertainties related to possible multiple states of hydrogen in STO^20–22^, we focused on substitutional nitrogen. Additionally, we examined the possibility for substitution/vacancy to be stabilized at a surface location. We employed large-scale first-principles calculations, which ensured high accuracy of the predicted electronic structure and bandgaps^23–32^.

We verified theoretical calculations by experimental studies of the optical properties of STO films, which were doped in-situ with nitrogen or hydrogen, or contained oxygen vacancies^32–36^. Contrary to often employed long-lasting high-temperature processing, the films were prepared without such processing. To ensure substitution locations inside a crystal, we grew single-crystal epitaxial films, whereas to introduce locations at boundaries, we deposited polycrystalline films.

Our observations suggest that boundary or surface locations for substitution/vacancy are more energetically favourable than locations inside the crystal. Deep in-gap states were theoretically predicted for substitutions inside crystals and experimentally detected in epitaxial films, whereas such states were absent for boundary locations as verified in polycrystalline films. Broad in-gap donor bands were found for oxygen vacancies. For all cases, only wide-bandgap insulator phases and bandgap widening were experimentally evidenced. The observations of wide-bandgap phases agree with theoretical predictions, and, furthermore, the calculations reveal some widening of bandgap for systems with in-gap defect states.

## Results and discussion

First, we summarize the results of simulations and discuss the properties of the neutral oxygen vacancies and nitrogen substitution atoms in non-magnetic STO. Both of these defects are characterized by unpaired spins. In calculations of such defects, all electrons are divided into two groups: those with spins up (alpha-electrons) and those with spins down (beta-electrons). The total spin of a defect is defined by the difference in the numbers of alpha- and beta-electrons. The computational details are presented in “Methods” section. Compared to the stoichiometric number of oxygen atoms, the concentration of the defects was 4.17% in the bulk crystal, 12.5% in the layer with defect in the thin-film slab, or 3.125% with respect to the whole slab.

### Oxygen vacancies

The oxygen vacancy is a common point defect in perovskite oxides. The majority of ab initio calculations conventionally focused on vacancies in the bulk STO^37–43^, with a few studies considering surface defects^41–44^. However, most of these calculations were performed within plane wave basis set using GGA + U approximation, which depends on the parameter choice. Here we investigated neutral vacancies V_O_ placed not only inside ultrathin films, but also on the surface, using the identical, parameter free, approach with full structures optimization. As a model, we used free standing symmetric 2D slabs built of 5 atomic layers and having similar TiO_2_–TiO_2_ terminations on both sides. The two unpaired electron spins arise after the removal of an oxygen atom. The spins are distributed among Ti atoms, which are not necessarily the nearest ones to the vacancy, as we noted before for BaTiO_3_^31^. An additional occupied local energy level or a band emerge in the band gap due to the formation of defects. The band appears because of interaction of periodically repeated defects.

**Figure 1:**
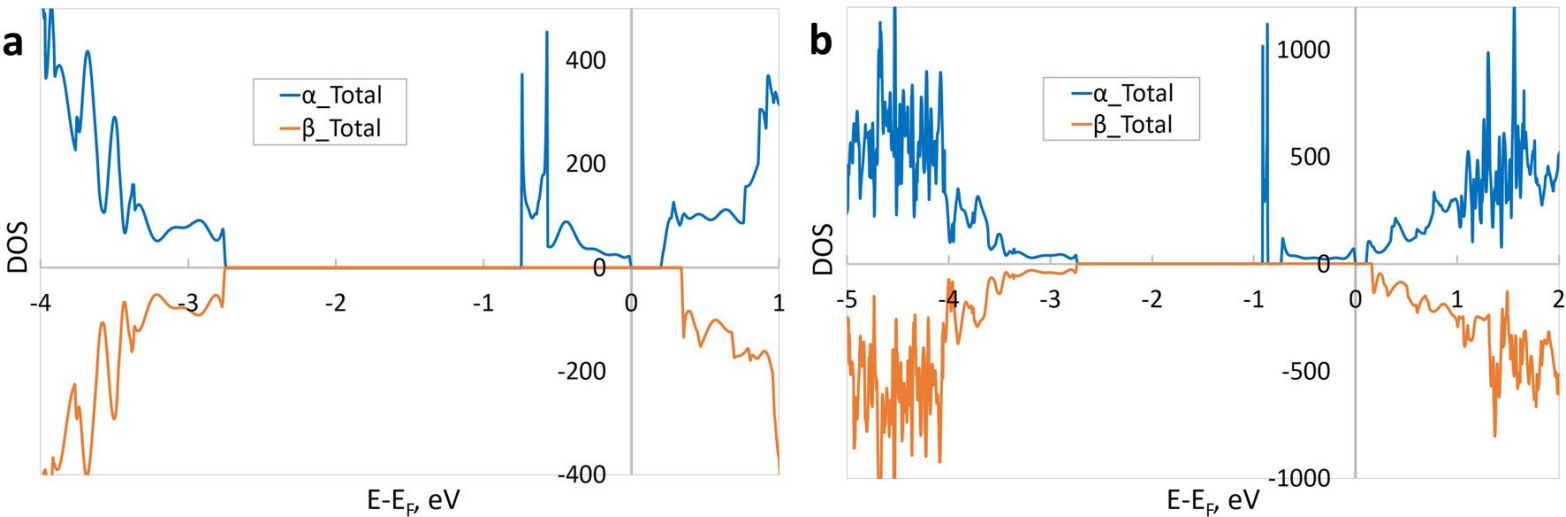
The electronic DOS for slabs with oxygen vacancy, total spin *S*_*z*_ = 1. (**a**) V_O_ in the central layer of the slab (space group SG 1); (**b**) V_O_ on the surface of the slab (SG 6). α_Total, β_Total—total DOS for alpha- and beta- (spin up/down) electrons; the zero value of the energy corresponds to the Fermi level.

First, we consider a neutral oxygen vacancy V_O_ in the central layer of the slab. The calculated density of states (DOS) for the total spin state *S*_*z*_ = 1 (Fig. 1a) reveals a modified bandgap, which is the direct transition for beta-electrons. The bandgap energy is *E*_*g*_ = 3.1 eV, larger than *E*_*g*_ = 2.5 eV in the 5-layer pristine slab with TiO_2_ terminations. Compared to an isolated energy level in the limit of a single defect, we observe a relatively broad donor defect band in the periodic model. The minimum energy difference between the bottom of the conduction band and the top of the in-gap vacancy band, or the defect ionization energy, is found to be *E*_*ion*_ = 0.2 eV. The corresponding transition is indirect for alpha-electrons.

The formation energy for the vacancy is defined by

1 $${E_{vac}} = E({V_O}) \, + E\left( O \right) - E\left( {perfect} \right),$$

where *E*(V_O_) and *E*(perfect) are the total energies of the system with and without vacancy, respectively, and *E*(O) is the half energy of a gas-phase O_2_ molecule (that is, O atom) in the triplet state. The calculated formation energy is *E*_*vac*_ = 6.7 eV. It should be noted that the energies *E*_*ion*_ and *E*_*vac*_ do not vary for systems with either *S*_*z*_ = 0 or *S*_*z*_ = 1.

Next, we inspect a neutral vacancy on the TiO_2_ surface of the slab. The calculations (Fig. 1b) give a bandgap energy *E*_*g*_ = 2.9 eV (the direct transition for beta-electrons) and an ionization energy *E*_*ion*_ = 0.1 eV (indirect transition for alpha-electrons), with a rather delocalized electron density around the vacancy. The defect formation energy is *E*_*vac*_ = 5.9 eV. Again, we note that *E*_*ion*_ and *E*_*vac*_ do not depend on the spin state of the system.

The calculations uncovered an interesting spin localization. For a vacancy in the centre of the slab (Fig. 1a), spins are localized on the Ti ions in the layer containing the vacancy. However, for a vacancy on the surface (Fig. 1b), spins are localized on the Ti ions in both the surface layer and central layer.

Overall, compared to the pristine slab, neutral vacancies slightly widen the bandgap and produce in-gap donor energy levels, at least by 0.1–0.2 eV below the conduction band. Importantly, the formation energy is by 0.8 eV smaller for the vacancy on the surface with respect to that inside the slab. This significant difference points to a preferential location of the vacancies at phase boundaries, interfaces, or surfaces in real systems.

### Substitutional nitrogen atoms

**Figure 2:**
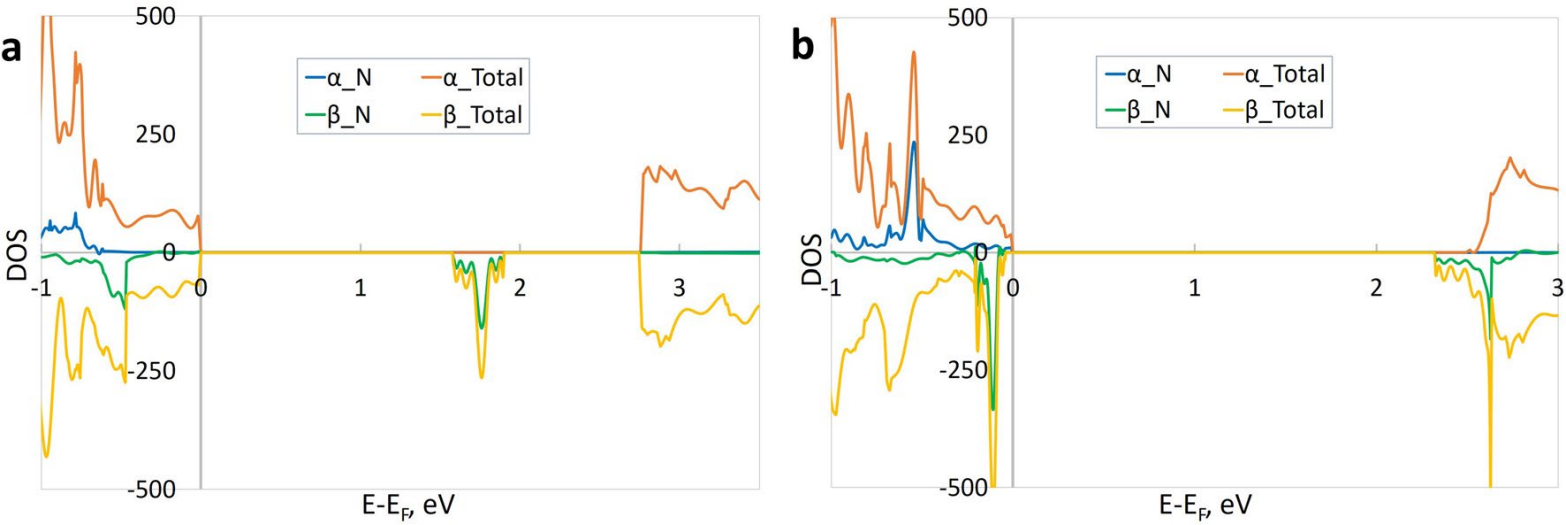
The electronic DOS for slabs with substitutional N atom. (**a**) N in the central layer of the slab (SG 25); (**b**) N on the surface of the slab (SG 6). α_Total, β_Total—total DOS for alpha- and beta-electrons, α_N, β_N—DOS projected onto N atom (alpha- and beta-electrons); the zero value of the energy corresponds to the Fermi level.

We modelled lattices with substitutional nitrogen atoms in three distinct locations: in the bulk STO (Supplementary Fig. S1), in the centre of the free standing slab (Fig. 2a), and lastly, on the surface of the slab (Fig. 2b).

For nitrogen in the bulk STO, the calculated direct bandgap for alpha-electrons is *E*_*g*_ = 3.0 eV, which is somewhat smaller than the gap *E*_*g*_ = 3.36 eV in the pristine cubic STO. Calculations reveal an unoccupied in-gap defect energy level, which is mainly produced by the nitrogen atom. The minimum energy difference between the lowest defect levels and the top of the valence band, or optical absorption energy here, is *E*_*abs*_ = 1.5 eV (indirect transitions of beta-electrons).

The defect formation energy *E*_*dop*_ is defined by

2 $${E_{dop}} = E\left( {{N_{dop}}} \right) - E\left( N \right) \, + E\left( O \right) - E\left( {perfect} \right),$$

where *E*(N_dop_) is the total energy of the crystal with nitrogen dopant and E(N) is the half energy of a gas-phase N_2_ molecule. The simulation of defective STO by SC 2 × 2 × 2 gives the energy *E*_*dop*_ = 5.7 eV.

Like nitrogen in the bulk STO, the nitrogen substitution in the centre of the slab also produces an unoccupied defect energy level in the band gap (Fig. 2a). The calculated bandgap energy is *E*_*g*_ = 2.8 eV for direct transition of alpha-electrons (cf. with 2.5 eV for pristine slab). The found absorption energy is *E*_*abs*_ = 1.6 eV for indirect transition of beta-electrons and the formation energy is *E*_*dop*_ = 5.5 eV.

Contrary to nitrogen in the bulk STO or inside the slab, substitutional atom on the surface of the slab does not create any defect levels in the band gap, but modifies only the edges of the valence and conduction bands (Fig. 2b). The modified bandgap is *E*_*g*_ ~ 2.4 eV, which is close to that in the pristine slab. The formation energy for nitrogen substitution on the surface of the slab is *E*_*dop*_ = 5.2 eV, which is by 0.3 eV smaller than substitution energy inside the slab. Thus, also substitutional nitrogen could prefer surface locations analogously to vacancies, although with a lesser energy gain.

It is worth emphasizing that the in-gap defect levels can be produced by the substitutional nitrogen located either in the slab or bulk, but not on the surface. This behaviour contrasts the presence of the in-gap levels for any location of the oxygen vacancy: either inside the slab or on its surface. Both the oxygen vacancies and substitutional atoms prefer surface locations over those inside the slabs.

For all defects, the wide bandgap insulator phases are predicted. Interestingly, compared to pure 5-layer STO slab with TiO_2_ terminations, in systems, where the in-gap states arise, the calculated band gaps are wider, by 0.6 eV (V_O_ in the central layer), 0.4 eV (V_O_ on the surface) and 0.3 eV (nitrogen in the central layer) respectively. At the same time, simulations reveal that the electronic transitions, related to the in-gap states, are indirect. According to the calculations, neither oxygen substitutions, nor vacancies can lead to often reported large band conductivity and significant optical absorption in the visible spectral range. To verify the theoretical predictions, we experimentally investigated the optical properties of STO films with oxygen substitutions or vacancies.

### Optical properties

Epitaxial and polycrystalline STO films were grown by pulsed laser deposition, during which anion dopants and/or oxygen vacancies were introduced *in-situ* by varying the ambient gas (“Methods”)^33^. The content δ of oxygen vacancies/substitutions was to ~ 0.3 in SrTiO_3-δ_X_δ_ (Supplementary section S1). The prepared films are marked here by S- and S*- for stoichiometric films, O- and O*- for oxygen deficient films, and N- and N*- (and H- and H*) for nitrogen-(hydrogen)-doped epitaxial and polycrystalline (*) ones, respectively. The optical properties were inspected by spectroscopic ellipsometry using very small intensity of light, that ensures probing of authentic, and not photoinduced properties.

**Figure 3:**
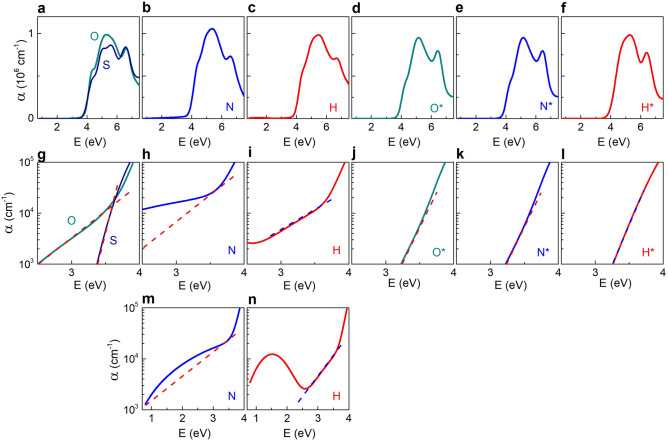
Absorption coefficient *α* as a function of photon energy. Spectral range is (**a**–**f**) from 0.7 to 7.5 eV, (**g**–**l**) from 2.5 to 4.0 eV, and (**m**,**n**) from 0.7 to 4.0 eV. Dashed lines show Urbach absorption in (**g**–**n**). STO films are marked as follows: *S* epitaxial stoichiometric, *O* epitaxial with oxygen vacancies, *N* epitaxial with nitrogen substitution, *H* epitaxial with hydrogen substitution, O* polycrystalline with oxygen vacancies, N* polycrystalline with nitrogen substitution, H* polycrystalline with hydrogen substitution.

All films exhibited profound absorption peaks centred at 5 eV, which is consistent with the fundamental absorption in regular STO (Fig. 3a–f).

In all films, a steep raise of the absorption coefficient *α* at photon energy *E* ~ 4 eV is caused by a direct band-to-band transition. We note that compared to the indirect bandgap in pure unstressed cubic STO, the theoretically predicted bandgaps are direct in the presence of oxygen vacancies/substitutions (see above) or epitaxial lattice strain^45^. The energy *E*_*d*_ of the direct bandgap was found using Tauc-type plots [(*αE*)^2^ ∝ (*E* – *E*_*d*_)] (Supplementary Fig. S2). The energy *E*_*0*_ of the lowest-energy critical point, corresponding to the strongest band-to-band transition, was extracted using second derivatives of the dielectric function (Supplementary Fig. S3). When compared to the lowest energy direct transition in bulk cubic STO, the energies *E*_*d*_ and *E*_*0*_ appeared to be clearly larger in the films (Table 1). The blueshift up to ~ 0.4 eV was detected in the polycrystalline doped N*- and H* films. The blueshift of ~ 0.2 eV was also found in stoichiometric epitaxial film (S), whereas it was absent in stoichiometric polycrystalline film (S*) (Supplementary Fig. S4).

**Table 1:** The bandgap (*E*_*d*_) and critical-point (*E*_*0*_) energies in different STO films and crystal.

	S	O	N	H	S*	O*	N*	H*	Crystal
*E*_*d*_, eV	3.98	4.00	4.03	4.06	3.85	3.94	4.15	4.18	3.85
*E*_*0*_, eV	4.07	4.08	4.10	4.13	3.90	4.01	4.22	4.26	3.80

As theoretically demonstrated, the lattice strain can lift or lower the conduction band^45^, whereas oxygen substitutions/vacancies can widen the bandgap (as shown in this work). Both mechanisms coexist and are difficult to separate in the epitaxial O-, N-, and H-films. However, the behaviour of the polycrystalline S*-, O*-, N*-, and H*-films points to the bandgap widening due to oxygen substitutions/vacancies. Importantly, our observations imply that commonly believed bandgap narrowing is very unlikely for several percent of oxygen substitutions /vacancies in STO.

Next, we closer inspected the absorption coefficient *α* at photon energies *E* < 4 eV. Semilog (*α*–*E*) plots (Fig. 3g–l) revealed the presence of an Urbach-type absorption^46^ [*α* ∝ exp(*E*)] in all films. Previously, we suggested that Fröhlich-type electron–phonon interactions may lead to steep Urbach tails in pure STO films^47^. This mechanism may also explain the behaviour here.

The Urbach-type absorption is superimposed with an additional contribution in the epitaxial N- and H-films (Fig. 3m,n). The additional peak at ~ 2 eV in the N-film and the peak at ~ 1.5 eV in the H-film evidence the presence of anion-specific deep in-gap states in these films. According to the calculations, these observations indicate substitutional locations inside the crystal for the epitaxial films. Importantly, the N- and H-specific absorption peaks were not detected in the polycrystalline N*- and H*-films. Because in-gap states were not expected for boundary substitutional locations, the difference between the epitaxial and polycrystalline films proves that anionic dopants are mainly located at phase boundaries, and not inside crystallites, in the polycrystalline films.

In the visible spectral range (photon energies ~ 1.8–3.1 eV), the absorption coefficient is *α* < 10^4^ cm^−1^ in the epitaxial O-, N-, and H-films and *α* < 10^3^ cm^−1^ in the rest of the films. This small magnitude of *α* indicates that visual observations of changes in colour and/or transmittance, that are often reported for long-lasting high-temperature processing of STO at reduced oxygen pressure or in the presence of hydrogen or nitrogen^9–16^, can be hardly caused by oxygen substitutions/vacancies. We stress that here we avoided such high-temperature processing, during which structural and/or compositional inhomogeneity, as well as networks of dislocations or other structural defects can easily form in STO^19^.

**Figure 4:**
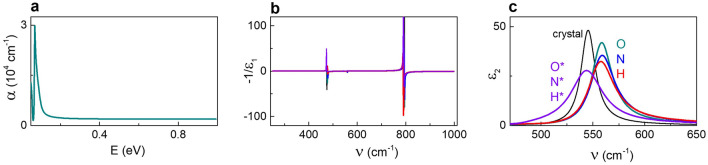
Optical properties in the infrared spectral range. (**a**) Absorption coefficient, (**b**) dielectric loss function, and (**c**) imaginary part of the dielectric function as a function of (**a**) photon energy or (**b**,**c**) wavenumber in different STO films. Data for all films overlap in (**a**,**b**). Data for reference crystal are shown in (**c**) for comparison.

For the photon energies *E* < 1 eV, spectral features related to the presence of in-gap states or free carriers were not detected in any of the films (Fig. 4a). The dielectric and loss functions in the infrared range (Fig. 4b,c) were consistent with regular STO behaviour and indicated lattice vibrations at approximately 59, 68, and 98 meV (475, 550, 790 cm^−1^)^48^. The strongest lines at ~ 550 cm^−1^ (Fig. 4c) correspond to transverse optical phonons present both in the cubic and tetragonal phases of STO. The lines at ~ 475 and 790 cm^−1^ (Fig. 4b) are related to longitudinal optical phonons.

Thus, our theoretical calculations and experimental observations are in excellent agreement with each other and demonstrate that the substitution of several percent of oxygen atoms with nitrogen, hydrogen, and/or vacancies leads to the wide-bandgap insulator phases for all substitution types and locations in STO. The detected anion-specific absorption in the epitaxial N- and H-films evidences the presence of in-gap states and is consistent with the theoretical picture of substitutional locations inside the crystals.

The obtained results imply that the bandgap narrowing and semiconductor-like band conductivity cannot be caused by oxygen substitutions/vacancies in STO. Contrary to the previous expectations of bandgap narrowing, our theoretical calculations and experimental observations point to the bandgap widening. Furthermore, the minimum calculated energy for carriers’ excitation from the in-gap states to the conduction band is at least 0.1 eV for oxygen vacancies, whereas other defects produce deeper states. Although the magnitude of 0.1 eV is small compared to the bandgap in STO, and the term “shallow” is often used to describe such in-gap states in STO, this magnitude is generally rather large. For instance, a gap of only 20–50 meV is associated with high-resistivity insulator phase in perovskite nickelates. Our observations suggest a minor if any carriers’ excitation at room-temperature in STO films.

Finally, we note that semiconductor-like behaviour is usually reported for STO subjected to a high-temperature processing for several hours to days. Our work evidences the lack of semiconductor-like properties in the absence of such processing. Concurrently, in the last years, it has been found that structural or compositional defects, other than oxygen vacancies/substitutions, can drive the electronic properties of STO towards semiconductor state^49–54^. We believe that during high-temperature processes, such defects can unintentionally form and lead to the increased optical absorption and electrical conductivity.

## Conclusions

Substitutions of several percent of oxygen atoms with nitrogen, hydrogen, or vacancies were studied in perovskite oxide SrTiO_3_ films. The large-scale theoretical first-principles calculations of the atomic and electronic structure of the crystals and ultrathin films were combined with experimental investigations of the optical properties of in-situ prepared epitaxial and polycrystalline films. It was theoretically predicted and experimentally verified, that the location of the substitutions and vacancies is more energetically favourable at phase boundaries, interfaces, or surfaces than inside a crystallite. Deep in-gap states were detected for substitutional atoms only inside crystallites, whereas in-gap donor bands were suggested for all locations of oxygen vacancies. The theoretical calculations and experimental observations evidenced wide-bandgap insulator phases for all cases. Furthermore, a tendency to bandgap widening was observed.

## Methods

### First-principles calculations

Ab initio first-principles computer simulations were performed to investigate neutral oxygen vacancies and substitutional nitrogen atoms in both bulk STO crystals and ultrathin freestanding STO (001) films. The structural and electronic properties of these systems, as well as the formation energy of the defects were examined within the linear combination of atomic orbitals approximation. We employed the B1WC advanced hybrid exchange–correlation functional of the density-functional-theory (DFT), as implemented in the CRYSTAL17 computer code^23^. Basis sets with Hay and Wadt small core pseudopotential were used for Sr and Ti atoms^24^, and the all-electron basis sets were applied for the description of oxygen atoms^25^ and nitrogen atoms^26^. The periodic supercell (SC) approach was employed to simulate point defects. More computational details can be found in our previous studies of perovskite (Ba,Sr)TiO_3_ and (Ba,Ca)TiO_3_ solid solutions^27–29^ and BaTiO_3_/SrTiO_3_ heterostructures^28,30^.

For analysis of substitutional nitrogen atom in the bulk STO crystal, we used SC with extended lattice constants 2 × 2 × 2 (40 atoms). In turn, to simulate thin STO films, we employed free-standing slabs in the form of 2D supercells. The surface parameter extension was 2 × 2 along *x* and *y* directions of the slab, which contained 5-layers (001) along the *z* axis and possessed TiO_2_ terminations on its both sides (52 atoms). The defects were then placed either in the central layer of the slab or on its surface.

Various possible spin states were considered by means of unrestricted open shell DFT calculations^23^. When an oxygen atom is removed from STO and a neutral vacancy V_O_ is formed, the spins of the two unpaired electrons are distributed among the Ti atoms near the vacancy. This system may have two different spin states: either *S*_*z*_ = 0 (the singlet state; two antiparallel spins) or *S*_*z*_ = 1 (the triplet state; two spins are parallel)^31,32^. Concurrently, the removal of an O^2−^ anion produces a charged oxygen vacancy V_O_^2+^, around which all chemical bonds are closed^32^. Nitrogen atom, which substitutes oxygen in STO, has one unpaired electron (here, N atom mimics missing O^2−^ ion) and, hence, the spin is *S*_*z*_ = 1/2.

An important issue is related to the symmetry of the crystal or film containing point defects. Here, the defects—substitutional nitrogen atoms and oxygen vacancies—were created in the SC constructed from a cubic STO (space group SG 221) with the Wickoff positions of atoms as follows: Sr:1b(1/2,1/2,1/2), Ti:1a(0,0,0), O:3d(1/2,0,0). The symmetry of the system is lowered upon the formation of a defect. Because the result of a full structural optimization depends on the symmetry of the system, the choice of symmetry affects the total energy, spin distribution, and the formation energy of defects, along with other important parameters. In turn, the symmetry is affected by both the dimension of the SC and the exact position of the defect. For example, the presence of defect may lower the cubic SG 221 symmetry of SC 2 × 2 × 2 down to tetragonal SG 99 or orthorhombic SG 25. Additionally, different atom spins can further lower the symmetry. Here, for all systems, we performed calculations with the lowest symmetry for each SC or, even, without any symmetry (SG 1). In this approach, artificial constraints imposed by symmetry are minimal or completely absent and the results only depend on atomic interaction potentials. Unfortunately, the computational cost of such calculations strongly increases.

### Experimental

Thin STO films (thicknesses of 80–100 nm) were grown by pulsed laser deposition, during which anion dopants and/or oxygen vacancies were introduced *in-situ* by varying the ambient gas^33^. Epitaxially polished (001) (La_0.3_Sr_0.7_)(Al_0.65_Ta_0.35_)O_3_ substrates (LSAT) and silicon substrates covered with native surface oxide (Si/SiO_2_) were used to prepare epitaxial and polycrystalline films, respectively. The films were deposited in pairs (one on LSAT and another one on Si/SiO_2_) within a single process, thus ensuring similar chemical composition for both films in each pair. The different microstructures of these films were determined by the underlying substrates.

The spectroscopic ellipsometry measurements were performed using J. A. Woollam ellipsometers at room temperature. The measurements at photon energies from 0.75 to 8.8 eV were carried out on a VUV ellipsometer in dry nitrogen atmosphere and those at the energies from 25 meV to 1.0 eV were performed on an IR ellipsometer. The data were processed using a commercial WVASE32 software package. The dielectric function and optical constants (index of refraction, extinction coefficient, and absorption coefficient) were determined as a function of photon energy. Specific methodological aspects were presented in our previous studies^35,36^.

## Supplementary Information

Supplementary Information.

## Data Availability

The datasets generated and/or analyzed during the current study are available from the authors on reasonable request.
