# Supplementary material for: The electronic properties of SrTiO3-δ with oxygen vacancies or substitutions
Source: Sci Rep. 2021 Dec 2;11:23341. doi: 10.1038/s41598-021-02751-9 (PMC8639995; doi:10.1038/s41598-021-02751-9)
Supplement: Supplementary file 1 — Supplementary Information. [file 41598_2021_2751_MOESM1_ESM.pdf]

Supplementary Information

to

The electronic properties of $SrTiO_{3-\delta}$ with oxygen vacancies or substitutions

L. L. Rusevich,[1]* M. Tyunina,[2,3]** E. A. Kotomin,[1,4] N. Nepomniashchaia,[3] A. Dejneka[3]

[1] Institute of Solid State Physics, University of Latvia, Kengaraga Str. 8, LV-1063 Riga, Latvia
[2] Microelectronics Research Unit, Faculty of Information Technology and Electrical Engineering, University of Oulu, P. O. Box 4500, FI-90014 Oulu, Finland
[3] Institute of Physics of the Czech Academy of Sciences, Na Slovance 2, 18221 Prague, Czech Republic
[4] Max Planck Institute for Solid State Research, Heisenberg Str. 1, Stuttgart D-70569, Germany

S1. Oxygen vacancies/substitutions

S2. Theoretical analysis

S3. Optical properties

S1. Oxygen vacancies/substitutions

In cube-on-cube-type epitaxial STO films on LSAT substrates, the substrate-induced compressive in-plane misfit strain determines out-of-plane orientation of elastic tensors, which are associated with oxygen substitutions such as vacancies or dopants [1-4]. This peculiar effect allows for estimation of the vacancy/substitution concentration N_D as $N_D \approx (c_{11}/D)\cdot s_D$, where $c_{11} = 3.48\times10^{11}$ N/m^2 is the elastic constant of STO, $D \approx (2\ldots5)$ eV is the elastic tensor component, and s_D is the defect-induced out-of-plane chemical strain [4-6]. The strain s_D was determined from the measured lattice parameters as described before [4]. The estimated concentration was in the range of $\sim(0.5\ldots1.5)\times10^{28}$ m^{-3}. This estimation suggests large content δ of oxygen substitutions X to $\delta \approx 0.3$ in the films of $SrTiO_{3-\delta}X_\delta$. This content agreed with the content of nitrogen (hydrogen) assessed by secondary ion mass spectroscopy (SIMS) on a TOF-SIMS5 IONTOF instrument at CEITEC, Brno, Czech Republic [7].

[1] M. Tyunina et al., *Phys. Rev. Research* **2**, 023056 (2020).
[2] M. Tyunina et al., *APL Materials* **8**, 071107 (2020).
[3] M. Tyunina et al., *Phys. Chem. Chem. Phys.* **22**, 24796 (2020).
[4] M. Tyunina et al., *J. Mater. Chem. C* **9**, 1693 (2021).
[5] E. Clouet, C Varvenne, T. Jourdan, *Comput. Mater. Sci.* **147,** 49 (2018).
[6] D. A. Freedman, D. Roundy, T. A. Arias, *Phys. Rev. B* **80**, 064108 (2009).
[7] https://www.ceitec.eu/secondary-ion-mass-spectroscopy-ion-tof-tof-sims5/e1360

S2. Theoretical analysis

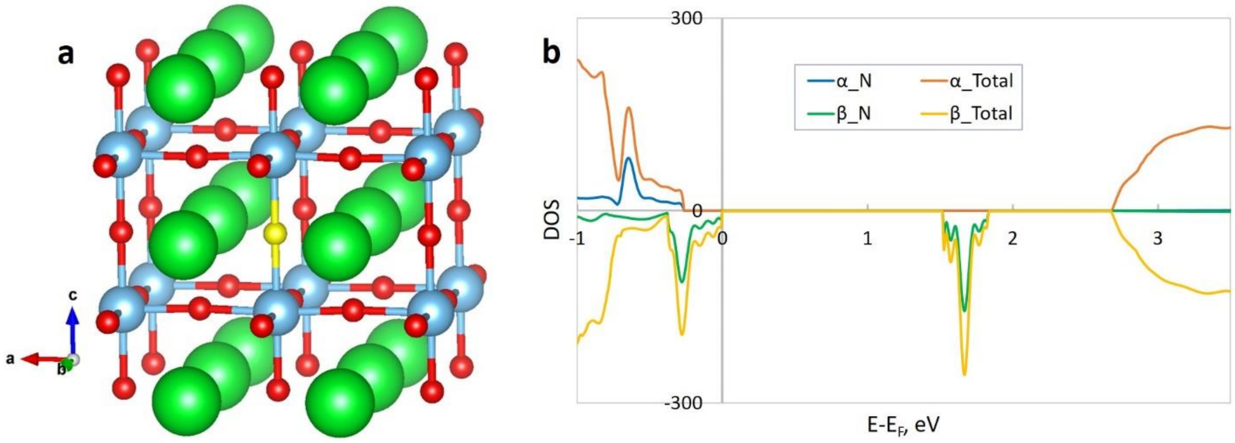

Fig. S1. N substitutional atom in bulk STO crystal. (**a**) full-relaxed system, SG 25 (yellow ball shows N, blue — Ti, green — Sr, red — oxygen); (**b**) the electronic DOS for this system. α_Total, β_Total — total DOS for alpha- and beta-electrons, α_N, β_N — DOS projected onto N atom (alpha- and beta-electrons); the zero value of the energy corresponds to the Fermi level.

S4. Optical properties

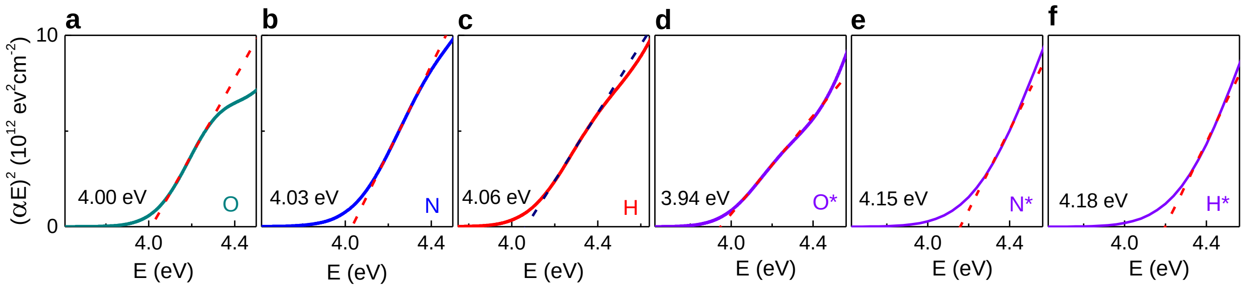

Fig. S2. Tauc-type plots for direct transition in the (**a-c**) STO/LSAT and (**d-f**) STO/Si films.

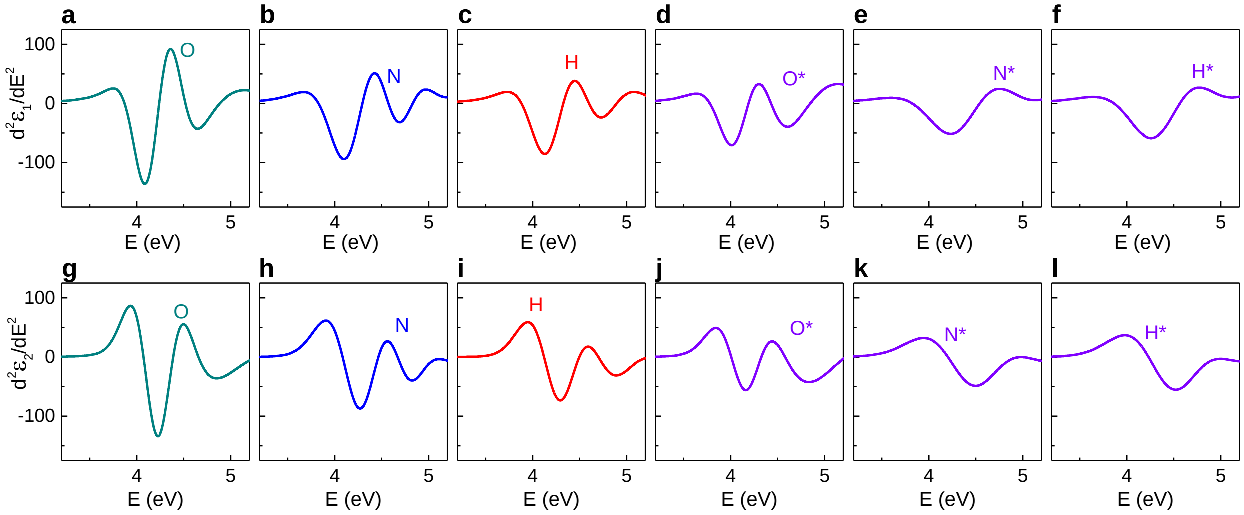

Fig. S3. Second derivatives of the (**a-f**) real and (**g-l**) imaginary parts of the dielectric function in the (**a-c**, **g-i**) epitaxial and (**d-f**, **j-l**) polycrystalline STO films.

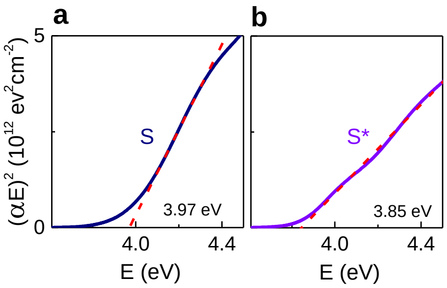

Fig. S4. Tauc-type plots for direct transition in the stoichiometric (**a**) STO/LSAT and (**b**) STO/Si films.
